# Risk factors of lymph node metastasis or lymphovascular invasion for early gastric cancer: a practical and effective predictive model based on international multicenter data

**DOI:** 10.1186/s12885-019-6147-6

**Published:** 2019-11-06

**Authors:** Jian-Xian Lin, Zu-Kai Wang, Wei Wang, Jacopo Desiderio, Jian-Wei Xie, Jia-Bin Wang, Jun Lu, Qi-Yue Chen, Long-Long Cao, Mi Lin, Ru-Hong Tu, Chao-Hui Zheng, Ping Li, Amilcare Parisi, Zhi-Wei Zhou, Chang-Ming Huang

**Affiliations:** 1 Department of Gastric Surgery, Fujian Medical University Union Hospital, Fuzhou, 350001 Fujian People’s Republic of China; 2 Department of Gastric and Pancreatic Surgery, Sun Yat-sen University Cancer Center, Guangzhou, 510060 Guangdong People’s Republic of China; 3 Department of Digestive Surgery, St. Mary’s Hospital, University of Perugia, 05100 Terni, Italy

**Keywords:** Lymph node metastasis, Lymphovascular invasion, Early gastric cancer, Predictive model, Recursive partitioning analysis

## Abstract

**Background:**

Most lymph node metastasis (LNM) models for early gastric cancer (EGC) include lymphovascular invasion (LVI) as a predictor. However, LVI must be confirmed by postoperative pathology. In this study, we aimed to develop a model for predicting the risk of LNM/LVI in EGC using preoperative factors.

**Methods:**

EGC patients who underwent radical gastrectomy at Fujian Medical University Union Hospital and Sun Yat-sen University Cancer Center (*n* = 1460) were selected as the training set. The risk factors of LNM/LVI were investigated. Data from the International study group on Minimally Invasive surgery for GASTRIc Cancer trial (*n* = 172) were selected as the validation set.

**Results:**

In the training set, the incidence of LNM/LVI was 21.6%. The 5-year cancer-specific survival rates of patients with and without LNM/LVI were 92.4 and 95.0%, respectively, with significant difference (*P* = 0.030). Multivariable logistic regression analysis showed that the four independent risk factors for LNM/LVI were female, tumor larger than 20 mm, submucosal invasion and undifferentiated tumor histological type (all *P* <  0.05); the area under the curve (AUC) was 0.694 (95% confidence interval [CI]: 0.659–0.730). Patients were divided into low-risk, intermediate-risk, high-risk and extremely high-risk groups by recursive partitioning analysis; the incidences of LNM/LVI were 5.4, 12.6, 24.2 and 37.8%, respectively (*P* <  0.001). The AUC of the validation set was 0.796 (95%CI, 0.662–0.851) and the predictive performance of the LNM/LVI risk in the validation set was consistent with that in the training set.

**Conclusions:**

The risk of LNM/LVI in differentiated mucosal EGC is low, which indicated that endoscopic resection is a treatment option. The risk of LNM/LVI in undifferentiated mucosal EGC and submucosa EGC are high and gastrectomy with lymph node dissection is suggested.

## Background

With the advancement of diagnostic techniques and the popularization of health examinations, the diagnostic rate of early gastric cancer (EGC) worldwide has gradually increased [1]. Lymph node metastasis (LNM) is an important disease feature that affects the prognosis of patients with EGC and determines the extent of lymph node dissection. Japanese gastric cancer treatment guidelines suggest that the endoscopic resection (ER) of EGC is feasible [2]. Many scholars are also exploring ER for EGC, and from the earlier endoscopic mucosal resection (EMR) to the current endoscopic submucosal dissection (ESD), there has been an expanding trend of ER indications for EGC. The EMR/ESD indications were categorized as the absolute indication for standard EMR/ESD and the expanded indication for ESD [2, 3]. The absolute indication is mucosal EGC with differentiated type and size ≤2 cm. The following are expanded indications: (1) mucosal differentiated EGC without ulcers and size > 2 cm, (2) mucosal differentiated EGC with ulcers and size ≤3 cm, (3) differentiated EGC with submucosal invasion < 500 mm and size ≤3 cm, and (4) mucosal undifferentiated EGC without ulcers and size ≤2 cm. However, for patients with EGC who already have LNM, such treatment may risk postoperative tumor recurrence [4, 5]. Therefore, to explore the risk factors of LNM in EGC is of great clinical significance. However, most studies on the LNM of EGC include lymphovascular invasion (LVI) as one of the predictors [6, 7]. Since LVI must be confirmed by postoperative pathological examination, it is difficult to know the status of LVI preoperatively. The present of LVI usually indicates lymph node metastasis or micrometastasis [3, 8–11]. Current predictive models for a simultaneous assessment of LNM/LVI risk in EGC have not been reported. Therefore, this study explored the available preoperative factors for LNM/LVI in EGC and stratified the risk of LNM/LVI based on recursive partitioning analysis (RPA) using international multicenter data.

## Methods

### Patients

The clinicopathological data of patients who underwent radical gastrectomy from Fujian Medical University Union Hospital (FMUUH, January 1994 to December 2016) and Sun Yat-sen University Cancer Center (SYSUCC, January 1990 to December 2012) were retrospectively analyzed. The selection criteria were: (1) the depth of tumor invasion was confined to the mucosa or submucosa; (2) no distant metastasis; and (3) the number of lymph node harvested was > 15. Patients were excluded if they (1) had multiple primary cancers; (2) had received neoadjuvant therapy; or (3) had incomplete clinicopathological information. Finally, the study included 1460 patients. A total of 172 non-Asian subjects in the International study group on Minimally Invasive surgery for GASTRIc Cancer (IMIGASTRIC) trial from January 2000 to December 2014 were selected as the validation set. The median follow-up time in FMUUH was 57 months (5–259 months), the median follow-up time in SYSUCC was 58 months (1–209 months), and the median follow-up time in IMIGASTRIC was 69.5 months (1–176 months). The study was approved by the FMUUH, SYSUCC and St. Mary’s Hospital Institutional Review Board.

### Variables and definitions

Variables investigated included age, sex, tumor location, tumor size, depth of invasion, tumor histological types, and LNM/LVI. The best cutoff for tumor size was 20 mm according to the maximum of the Youden index. The depth of invasion was divided into mucosal and submucosal invasion according to the eighth edition of American Joint Committee on Cancer (AJCC) gastric cancer staging system [12]. Lesions limited to the mucosa or submucosa were defined as early gastric cancer, with or without local lymph node metastasis [13]. According to the Japanese classification of gastric cancer, tumor histological types were classified into: (1) the differentiated histological type (papillary adenocarcinoma, well to moderately differentiated tubular adenocarcinoma) and (2) the undifferentiated histological type (poorly differentiated adenocarcinoma, mucinous adenocarcinoma, and signet ring cell carcinoma). LNM/LVI included LNM positive and LVI negative (LNM+/LVI-), LNM negative and LVI positive (LNM−/LVI+), and LNM positive and LVI positive (LNM+/LVI+).

### Follow-up

The follow-up policy at our center for the patients after surgery was every 3 to 6 months for the first 2 years consisted of clinic visits, with computed tomography (CT) and labs scans, and every 6 to12 months for following 3 to 5 years, then annually afterwards. Deaths because of cancer were recorded as events, and deaths secondary to other causes were censored.

### Statistical analysis

Chi-square test or Fisher’s exact test were used for analysis of categorical variables, whereas Mann-Whitney U test or Student’s t test were used for comparisons of continuous variables. The Kaplan-Meier method was used to estimate the cancer-specific survival (CSS). Uni- and multivariable logistic regression analysis were performed to identified the risk factors for LNM/LVI. The receiver operating characteristic (ROC) curves were plotted separately for the training and validation sets and the areas under the curve (AUC) were calculated. According to the results of multivariable analysis, RPA was used to divide the patients into different risk groups. In this study, different groups were obtained according to RPA, and groups with similar incidence of LNM/LVI were reintegrated into one risk group. Finally, four risk groups were obtained, and the incidence of LNM/LVI increased in turn, which were defined as: low risk group, intermediate-risk group, high-risk group and extremely high-risk group. A 1:3 propensity score matching ratio was set to minimize the differences between the training set and validation set due to age, depth of invasion, sex, tumor histological types and tumor size. All statistical analyses were performed using SPSS 22.0 (SPSS, Chicago, IL, USA) and R 3.4.2 (The R Foundation for Statistical Computing, Vienna, Austria). A *P* value < 0.05 was considered statistically significant and all statistical tests were two-sided.

## Results

### Clinicopathological characteristics of patients

**Fig. 1:**
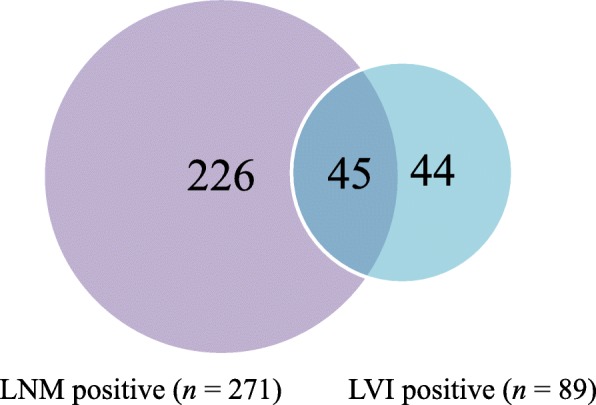
Venn diagram showing details of LNM/LVI. Values indicate number of patients

A total of 1632 patients with EGC were enrolled in this study, including 1460 cases in the training set and 172 cases in the validation set. The average age of patients in the training set was 57.43 ± 11.5 years, of which 815 (55.8%) were ≤ 60 years and 645 (44.2%) were > 60 years. The training set included 1024 males (70.1%) and 436 females (29.9%); there were 242 (16.6%) patients with tumor located in the upper region, 475 (32.5%) in the middle region, 643 (44.0%) in the lower region and 100 (6.8%) overlapped multiple regions; the average tumor size was 24.7 ± 15.4 mm, of which 850 (58.2%) had tumor size ≤20 mm and 610 (41.8%) had tumor size > 20 mm; there were 641 (43.9%) tumors confined to the mucosa and 819 (56.1%) infiltrating into the submucosa; a total of 502 (34.4%) differentiated tumors and 958 (65.6%) undifferentiated tumors were present; the average number of examined lymph nodes (No. of ELNs) was 28.70 ± 11.5; there were 1189 (81.4%) node-negative (N0) cases and 271 (18.6%) node-positive (N1) cases; most of the patients received D2 lymph node dissection (75.1%); a total of 1145 cases (78.4%) did not have LNM/LVI, and 315 cases (21.6%) presented with LNM/LVI. In the 315 cases with LNM/LVI, LNM−/LVI+ accounted for 44 cases (14.0%), LNM+/LVI- accounted for 226 cases (71.7%), and LNM+/LVI+ accounted for 45 cases (14.3%) (Fig. 1). The different clinicopathological characteristics between the training set and validation set are shown in Table 1.

**Table 1:** Clinicopathological characteristics of EGC patients in the training set and the validation set

Parameter	Training set	Validation set	*P-*value
	(*n =* 1460)	(*n =* 172)	
	*n* (%)	*n* (%)	
Age (mean ± SD)	57.43 ± 11.5	66.6 ± 10.6	< 0.001
Age			< 0.001
≤ 60	815 (55.8)	49 (28.5)	
> 60	645 (44.2)	123 (71.5)	
Sex			< 0.001
Male	1024 (70.1)	94 (54.7)	
Female	436 (29.9)	78 (45.3)	
Tumor location			0.004
Upper	242 (16.6)	28 (16.3)	
Middle	475 (32.5)	58 (33.7)	
Lower	643 (44.0)	86 (50.0)	
Overlap^a^	100 (6.8)	0 (0)	
Tumor size (mm, mean ± SD)	24.7 ± 15.4	27.8 ± 16.2	0.016
Tumor size			< 0.001
≤ 20 mm	850 (58.2)	67 (39.0)	
> 20 mm	610 (41.8)	105 (61.0)	
Depth of invasion			< 0.001
Mucosa	641 (43.9)	100 (58.1)	
Submucosa	819 (56.1)	72 (41.9)	
Tumor histological types			< 0.001
Differentiated	502 (34.4)	122 (70.9)	
Undifferentiated	958 (65.6)	50 (29.1)	
No. of ELNs (mean ± SD)	28.70 ± 11.5	25.70 ± 11.5	0.001
N stage			0.015
N0	1189 (81.4)	153 (89.0)	
N+	271 (18.6)	19 (11.0)	
Extent of lymphadenectomy			< 0.001
D1	155 (10.6)	28 (16.3)	
D1+	209 (14.3)	39 (22.7)	
D2	1096 (75.1)	105 (61.0)	
LNM/LVI			0.032
Absent	1145 (78.4)	147 (85.5)	
Present	315 (21.6)	25 (14.5)	

*Abbreviations*: *SD* standard deviation, *LNM* lymph node metastasis, *No. of ELNs* number of examined lymph nodes, *LVI* lymphovascular invasion

^a^Overlap, tumor invaded two or more regions simultaneously

### Relationship between LNM/LVI and 5-year cancer-specific survival

**Fig. 2:**
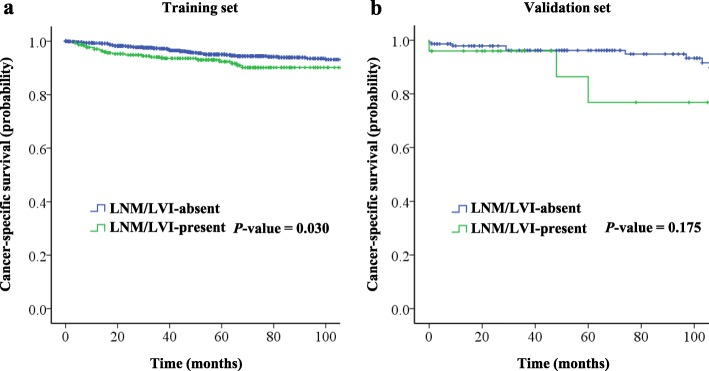
CSS of patients with EGC underwent radical gastrectomy between the LNM/LVI-absent and LNM/LVI-present groups. **a** In the training set. **b** In the validation set

In the training set, the 5-year CSS rate was 94.5%. For the LNM/LVI-present and LNM/LVI-absent groups, the 5-year CSS rates were 92.4 and 95.0%, respectively, with statistically significant difference (*P* = 0.030, Fig. 2a).

In the validation set, the 5-year CSS rate was 94.2%. The 5-year CSS rate in the LNM/LVI-absent group was 96.2%, which was higher than 76.8% in the LNM/LVI-present group, but not yet statistically significant (*P* = 0.175, Fig. 2b).

We also calculated the 5-year CSS rate of only LNM+ and only LVI+ groups in training set. The Kaplan-Meier analysis showed that in patients without LVI, the 5-year CSS rate in the LNM- group was significantly higher than that in the LNM+ group (94.9 vs. 91.8, *P* = 0.019); in patients without LNM, the 5-year CSS rate in the LVI- group was similar to the LVI+ group (95.0 vs. 97.5, *P* = 0.478) (Additional file 1: Figure S1).

### Univariable and multivariable analyses by using LNM/LVI or LNM as the outcome

Univariable analysis showed that LNM/LVI was closely related to sex, tumor size, depth of invasion and tumor histological types (all *P* <  0.05). Female, a tumor greater than 2 cm, invasion of the submucosa and an undifferentiated tumor were more likely to present with LNM/LVI. No significant correlation was evident between age or tumor location and LNM/LVI. Multivariable analysis showed that sex (odds ratio [OR] = 1.492, 95% confidence interval [CI]: 1.134–1.963, *P* = 0.004), tumor size (OR = 1.536, 95%CI: 1.184–1.992, *P* = 0.001), depth of invasion (OR = 2.898, 95%CI: 2.117–3.858, *P* <  0.001) and tumor histological types (OR = 1.983, 95%CI: 1.474–2.668, *P* <  0.001) were independent predictors for LNM/LVI (Table 2).

**Table 2:** Uni- and multivariable analysis for LNM/LVI of EGC patients in the training set

Parameters	Univariable Analysis		Multivariable Analysis	
	Odds Ratio (95%CI)	*P-*value	Odds Ratio (95%CI)	*P-*value
Age				
≤ 60	Ref			
> 60	0.997 (0.776–1.282)	0.984		
Sex				
Male	Ref		Ref	
Female	1.476 (1.135–1.92)	0.004	1.492 (1.134–1.963)	0.004
Tumor location		0.714		
Upper	Ref			
Middle	1.117 (0.765–1.63)	0.568		
Lower	1.001 (0.695–1.443)	0.994		
Overlap^a^	1.28 (0.739–2.217)	0.378		
Tumor size				
≤ 20 mm	Ref		Ref	
> 20 mm	1.73 (1.346–2.224)	< 0.001	1.536 (1.184–1.992)	0.001
Depth of invasion				
Mucosa	Ref		Ref	
Submucosa	2.939 (2.22–3.892)	< 0.001	2.898 (2.177–3.858)	< 0.001
Tumor histological types				
Differentiated	Ref		Ref	
Undifferentiated	2.03 (1.52–2.71)	< 0.001	1.983 (1.474–2.668)	< 0.001

*Abbreviations*: *Ref* reference, *CI* confidence interval, *LVI* lymphovascular invasion, *EGC* early gastric cancer, *LNM* lymph node metastasis

^a^Overlap, tumor invaded two or more regions simultaneously

Analysis of risk factors for LNM alone showed that tumor size, depth of invasion, tumor histological types and LVI were independent predictors for LNM. Sex was not an independent predictor for LNM (Additional file 2: Table S1).

### Validation of the multivariable regression model

**Fig. 3:**
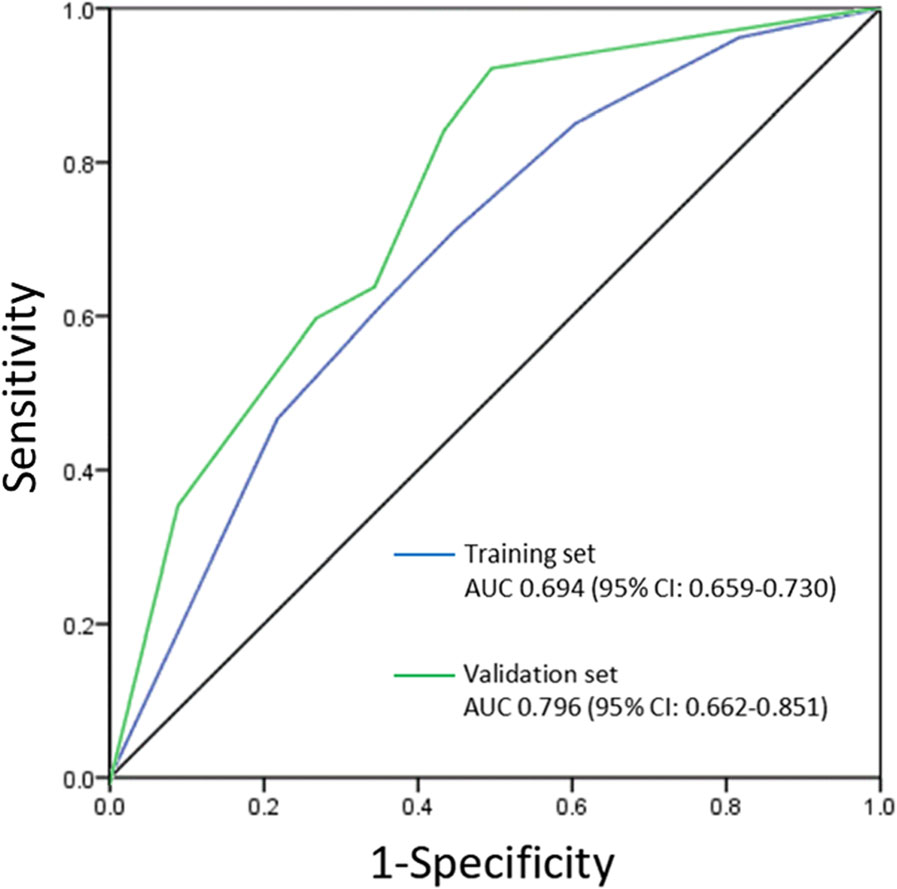
ROC curve of the multivariable model for predicting LNM/LVI in patients with EGC

The training set ROC curve and the validation set ROC curve (Fig. 3) were used to validate this multivariable regression model. In the training set, the AUC was 0.694 (95%CI: 0.659–0.730). In the validation set, the AUC was 0.796 (95%CI: 0.662–0.851). The model had moderate to good accuracy in both the training set and validation set.

### Risk groups of LNM/LVI according to RPA

**Fig. 4:**
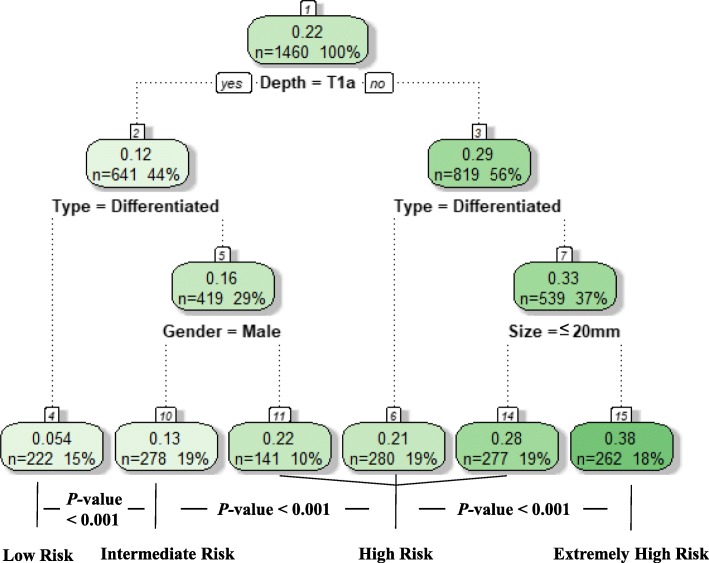
Classification tree for LNM/LVI status

**Fig. 5:**
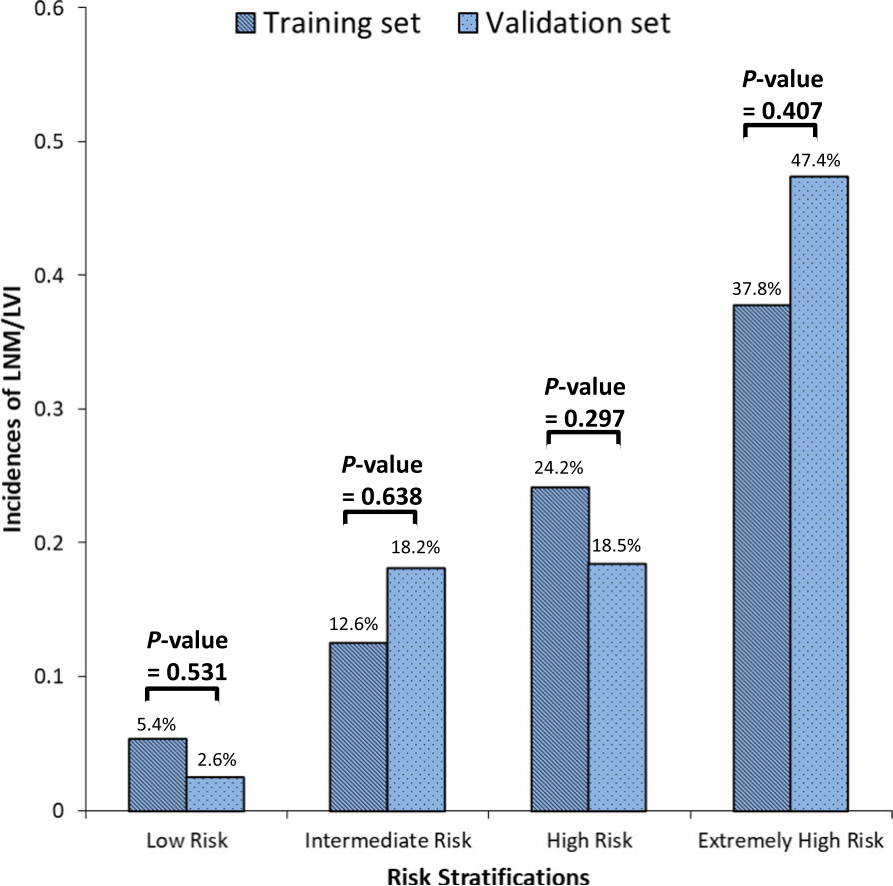
Incidences of LNM/LVI of the different study cohorts by risk groups

Based on the results of the multivariable analysis, RPA was performed to classify the patients into different risk groups. The four independent risk factors included in the RPA were sex, tumor size, depth of invasion and tumor histological types. According to the R software prioritization of binary variables, the group was divided into subgroups, and the patients in the training set were reclassified into 6 groups ultimately. Patients with similar incidences of LNM/LVI were pooled, and the patients were divided into low-risk, intermediate-risk, high-risk and extremely high-risk groups. In this training set, there were 222 (15%) low-risk patients (T1a, differentiated, regardless of tumor size and sex), 278 (19%) intermediate-risk patients (T1a, undifferentiated, male, regardless of tumor size), 698 (48%) high-risk patients (T1a, undifferentiated, female, regardless of tumor size; T1b, differentiated, regardless of tumor size or sex; T1b, undifferentiated, tumor size ≤20 mm, regardless of sex) and 262 (18%) extremely high-risk patients (T1b, undifferentiated, tumor size > 20 mm, regardless of sex). The incidences of LNM/LVI were 5.4, 12.6, 24.2 and 37.8% from low-risk to extremely high risk groups, respectively, with significant difference (all *P* <  0.001, Fig. 4). When the RPA model was applied to the validation set, the predictive performance of the LNM/LVI risk was consistent with that in the training set (all *P* > 0.1, Fig. 5).

### Propensity score matching analysis

We further used propensity score matching method to balance the differences in clinicopathological data between the training set and validation set, and try to validate the predictive model in the matching set. The propensity score matching ratio was set to 1:3 ratios to minimize the differences between the two groups due to age, depth of invasion, sex, tumor histological types and tumor size with the nearest neighbor method using R software. The training set and validation set were comparable in terms of age, sex, tumor size, depth of invasion, tumor histological types, N stage, LNM/LVI (all *P* > 0.05) (Additional file 2: Table S2). After matching, the incidences of LNM/LVI in the training set were 5.7, 15.2, 22.1 and 41.4% in low-risk, intermediate-risk, high-risk and extremely high-risk groups, respectively (*P*_trend_ <  0.001). The model’s prediction of the incidence of LNM/LVI in the training set and validation set were not statistically different between the different risk groups (all *P* > 0.1) (Additional file 2: Table S3).

## Discussion

Our study developed an LNM/LVI predictive model for EGC using four independent variables: sex, tumor size, depth of invasion and tumor histological types. The AUC in the training set and the validation set were 0.694 (95% CI: 0.659–0.730) and 0.796 (95%CI, 0.662–0.851) respectively. The incidences of LNM/LVI for low- to extremely high-risk patients were 5.4, 12.6, 24.2 and 37.8%, respectively (*P* <  0.001).

With the development of endoscopic technology and the increase in population aging in recent years, endoscopic resections, represented by EMR and ESD, to treat EGC have been widely carried out [14, 15]. Compared with traditional abdominal surgery, ER has many advantages such as less trauma and improved postoperative quality of life [16–19]. However, the endoscopic treatment of gastric cancer itself has its own limitations, namely, the inability of a surgeon to perform a dissection of the lymph nodes around the stomach where metastasis may occur. In addition, there is no effective or accurate way to predict LNM. Thus, endoscopic treatment is faced with a certain degree of a postoperative recurrence risk, and controversy exists [4, 5]. Therefore, a study to investigate the risk factors for potential LNM in EGC is important. Zheng et al. found that age, macroscopic types, tumor size, tumor histological types, the degree of differentiation, ulceration, LVI, and the depth of invasion were closely related to the LNM of EGC and thus established a nomogram to predict the risk of LNM in EGC [7]. Lee et al. conducted a study focusing on early gastric papillary adenocarcinoma and found that LVI was the only risk factor for LNM in EGC [20]. These studies confirm that LVI is an independent risk factor for LNM. Some studies even showed that LVI is associated with poor prognosis in patients with gastric cancer [21, 22]. In our study, the similar 5-year CSS rates of LVI+ and LVI- group may be due to the small proportion of LVI+ in patients without LNM. LVI sometimes indicates that patients have a significant risk of LNM and may lead to a worse prognosis. Moreover, it is suggested in the EMR/ESD-related guidelines that if the pathology confirms the presence of LVI in the resected specimen, the additional radical gastrectomy is needed [2]. From this point of view, LVI is of great significance for patients with gastric cancer, especially those with EGC who may receive ESD/EMR surgery. However, most of the previous studies on EGC LNM include LVI as one of the predictive risk factors, which makes these findings have obvious limitations in clinical application. On the other hand, in Kim et al.’s study, 53.4% (31/58) of EGC patients with LNM had LVI, while the other 46.6% did not have LVI in postoperative pathological examination [23]. In this study, as shown in Fig. 1, there were 45 of EGC patients with LNM had LVI (16.6%, 45/271); not all the EGC patients with LNM had LVI. While there were only 44 cases of N0 patients with LVI in this study. If LVI alone with N0 status was used to be an endpoint, there were 271 EGC patients with LNM will not be analyzed. Therefore, we combined LNM and LVI as an endpoint in order to make a more comprehensive assessment of the status of lymph node metastasis and lymphovascular invasion, and hope to provide more precisely medical evidences for the strategy choices of radical surgery for EGC.

Previous studies on the LNM of EGC mostly report that the tumor size, depth of invasion and tumor histological types have impacts on LNM [7, 24, 25]. However, whether sex has an impact on LNM in EGC remains to be determined. Pyo et al. established a model for predicting LNM in poorly differentiated-type intramucosal gastric cancer. The effect of sex on LNM was comparable with that of tumor size and depth of invasion [6]. However, in analysis of the risk factors of LNM in intramucosal EGC, Kim et al. found sex was not a risk factor [24]. The study by Deng et al. suggests that estrogen receptor-α, a mark highly correlated with LNM, is highly expressed in human gastric cancer [26]. We compared the incidence of LNM/LVI in women ≥60 years old and < 60 years old. The results showed that the incidence of LNM/LVI in women ≥60 years old was lower than < 60 (24.6% vs. 27.6%), but not yet statistically significant (*P* = 0.484). In addition, we compared the differences in the incidence of LNM/LVI between men and women in different age groups. The results showed that the incidence of LNM/LVI was significantly lower in men < 60 years old than in women (18.5 vs. 27.6, *P* = 0.004). While in patients ≥60 years old, the incidence of LNM/LVI in men was comparable to that of women (20.6% vs. 24.6%, *P* = 0.268) (Additional file 2: Table S4). These results indicate that the difference in the incidence of LNM/LVI between men and women disappears with age increasing, which may be caused by the regression of estrogen. Therefore, we hypothesize that the high incidence of LNM/LVI in female patients may be due to higher estrogen levels in female which has a direct or indirect impact on LNM or LVI. Based on the results of the multivariable analysis, a predictive model of LNM/LVI for EGC was established. The AUC of the model in the training set and the validation set were 0.694 and 0.796 respectively, which were no lower than that of the predictive model established by Pyo et al. (the AUC of the training set was 0.70, and the AUC of the internal validation set was 0.68) [27]. The results show that the model has moderate to good accuracy in both the training set and the validation set.

**Fig. 6:**
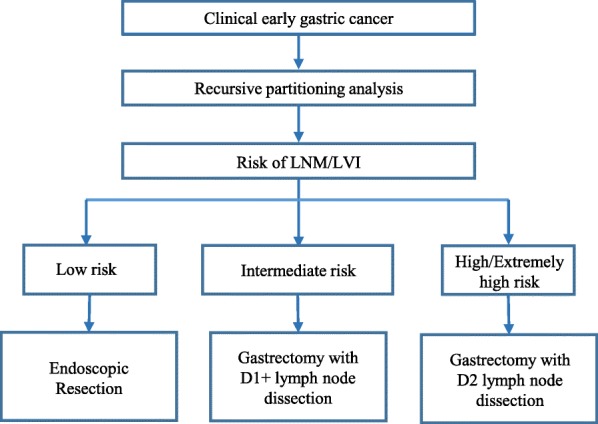
Proposed algorithm for the endoscopic or surgical treatment of early gastric cancer

Based on LNM/LVI status, we further performed a RPA to reclassify the patients into different risk groups then proposed strategies for lymph node dissection according to the risk group (Fig. 6). RPA is a statistical method for multivariable analysis, which divides group into subgroups according to the priority of several binary independent variables to correctly classify the members of a group and intuitively generate a concise decision tree to determine decision rules with higher sensitivity or specificity [28]. This approach is widely used in medical decision-making. In 1982 Goldman et al. became the first to use a RPA to establish a decision tree for the diagnosis of patients with acute chest pain [29]. Recently, Fonarow et al. used a RPA to successfully group in-patients with acute decompensated heart failure [30]. In this study, we found that LNM/LVI was present in 5.4% of patients with differentiated EGC in the mucosa, which is relatively safe for receiving ER. LNM/LVI was found more than 12% in male with undifferentiated mucosal EGC, suggesting that these patients should be treated with D1+ lymph node dissection. The incidence of LNM/LVI in the high-risk and extremely high-risk patient groups (female undifferentiated mucosal EGC and submucosa EGC) were above 24%. Therefore, the recommendation is that these groups of patients receive standardized D2 lymph node dissection.

This study had several limitations. First, this was a retrospective study, and bias inevitably existed in the data selection. Second, the tumor size and the depth of invasion in this study were confirmed by postoperative pathology. Although obtaining relatively accurate preoperative data by endoscopy or endoscopic ultrasonography is possible, bias may exist when compared with the postoperative pathological diagnosis. Third, this study lacked subjective diagnostic information, such as the macroscopic types of EGC. Fourth, due to the lack of records of postoperative adjuvant therapy in the SYSUCC and IMIGASTRIC databases, we were unable to provide information about postoperative adjuvant therapy. However, to our knowledge, this study was the first to explore the effect of preoperative factors on potential LNM in patients with EGC using international multicenter data. An effective multivariable prediction model for LNM/LVI was established and validated, and the use of RPA made the model more clinically useful. Based on the findings of this study, we have proposed recommendations for lymph node dissection in EGC for different risk groups, which will be of benefit to surgeons in making better clinical decisions.

## Conclusions

In this study, by using PRA to divide EGC patients into different risk groups, we have found that the incidence of LNM/LVI in differentiated mucosal EGC is low, which indicated that ER is a treatment option. The incidences of LNM/LVI in undifferentiated mucosal EGC and submucosa EGC are high and gastrectomy with lymph node dissection is suggested.

## Supplementary information

**Additional file 1: Figure S1.** The 5-year CSS rate of only LNM+ and only LVI+ groups in the training set. a. In the LVI- group, compared the 5-year CSS rate between LNM+ and LNM- group. b. In the LNM- group, compared the 5-year CSS rate between LVI+ and LVI- group.

**Additional file 2: Table S1.** Uni- and multivariable analysis for LNM of EGC patients in the training set. **Table S2.** Clinicopathological characteristics of EGC patients in the training set and the validation set after 1:3 propensity matching. **Table S3.** Comparison of the incidences of LNM/LVI between different risk groups in the training set and validation set after a 1:3 matching. **Table S4.** Comparison of the incidences of LNM/LVI between age groups in male and female.

## Data Availability

The datasets used and/or analyzed during the current study are available from the corresponding author on reasonable request.

## Abbreviations

AJCC: American Joint Committee on Cancer
AUC: The areas under the curve
CI: Confidence interval
CSS: Cancer-specific survival
CT: Computed tomography
EGC: Early gastric cancer
EMR: Endoscopic mucosal resection
ER: Endoscopic resection
ESD: Endoscopic submucosal dissection
FMUUH: Fujian Medical University Union Hospital
IMIGASTRIC: The International study group on Minimally Invasive surgery for GASTRIc Cancer
LNM: Lymph node metastasis
LVI: Lymphovascular invasion
OR: Odds ratio
ROC: The receiver operating characteristic
RPA: Recursive partitioning analysis
SYSUCC: Sun Yat-sen University Cancer Center
