# Supplementary figures and images for: Risk factors of lymph node metastasis or lymphovascular invasion for early gastric cancer: a practical and effective predictive model based on international multicenter data
Source: BMC Cancer. 2019 Nov 6;19:1048. doi: 10.1186/s12885-019-6147-6 (PMC6836519; doi:10.1186/s12885-019-6147-6)

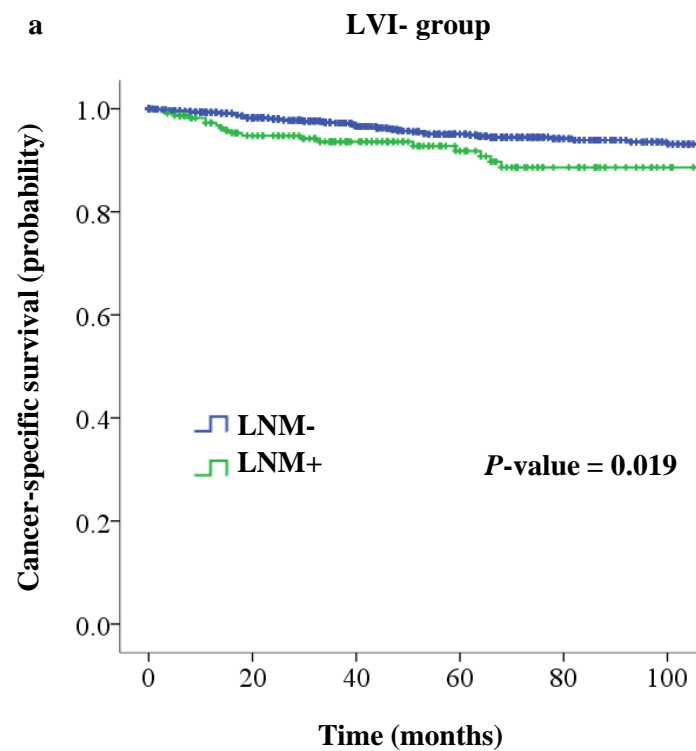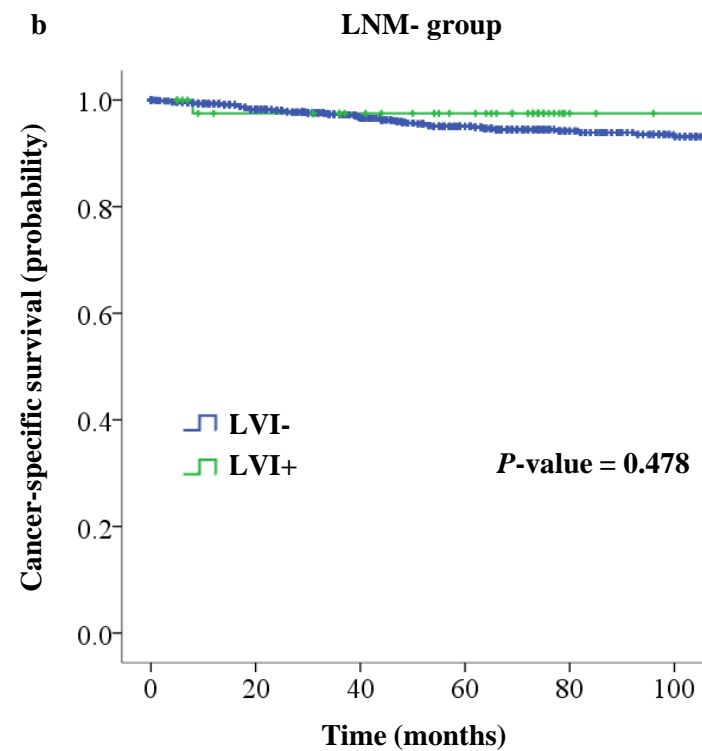

Supplement: Supplementary file 1 — Additional file 1: Figure S1. The 5-year CSS rate of only LNM+ and only LVI+ groups in the training set. a. In the LVI- group, compared the 5-year CSS rate between LNM+ and LNM- group. b. In the LNM- group, compared the 5-year CSS rate between LVI+ and LVI- group. [file 12885_2019_6147_MOESM1_ESM.pdf]
