# Supplementary material for: Risk factors of lymph node metastasis or lymphovascular invasion for early gastric cancer: a practical and effective predictive model based on international multicenter data
Source: BMC Cancer. 2019 Nov 6;19:1048. doi: 10.1186/s12885-019-6147-6 (PMC6836519; doi:10.1186/s12885-019-6147-6)
Supplement: Supplementary file 2 — Additional file 2: Table S1. Uni- and multivariable analysis for LNM of EGC patients in the training set. Table S2. Clinicopathological characteristics of EGC patients in the training set and the validation set after 1:3 propensity matching. Table S3. Comparison of the incidences of LNM/LVI between different risk groups in the training set and validation set after a 1:3 matching. Table S4. Comparison of the incidences of LNM/LVI between age groups in male and female. [file 12885_2019_6147_MOESM2_ESM.docx]

Table S1. Uni- and multivariable analysis for LNM of EGC patients in the training set

| Parameters | Univariable Analysis | |  | Multivariable Analysis |  |
| --- | --- | --- | --- | --- | --- |
|  | Odds Ratio (95%CI) | *P*-value |  | Odds Ratio (95%CI) | *P*-value |
| Age |  |  |  |  |  |
| ≤ 60 | Ref |  |  |  |  |
| > 60 | 0.851 (0.651-1.112) | 0.234 |  |  |  |
| Gender |  |  |  |  |  |
| Male | Ref |  |  | Ref |  |
| Female | 1.401 (1.056-1.86) | 0.019 |  | 1.252 (0.937-1.672) | 0.128 |
| Tumor location |  | 0.551 |  |  |  |
| Upper | Ref |  |  |  |  |
| Middle | 1.17 (0.788-1.737) | 0.437 |  |  |  |
| Lower | 0.939 (0.639-1.38) | 0.749 |  |  |  |
| Overlap* | 0.988 (0.539-1.81) | 0.968 |  |  |  |
| Tumor size |  |  |  |  |  |
| ≤ 20mm | Ref |  |  | Ref |  |
| > 20mm | 2.274 (1.653-3.127) | < 0.001 |  | 1.9 (1.362-2.649) | < 0.001 |
| Depth of invasion |  |  |  |  |  |
| Mucosa | Ref |  |  | Ref |  |
| Submucosa | 2.777 (2.063-3.738) | < 0.001 |  | 2.467 (1.807-3.352) | < 0.001 |
| Tumor histological types |  |  |  |  |  |
| Differentiated | Ref |  |  | Ref |  |
| Undifferentiated | 2.246 (1.639-3.077) | < 0.001 |  | 2.196 (1.58-3.052) | < 0.001 |
| LVI |  |  |  |  |  |
| Absent | Ref |  |  | Ref |  |
| Present | 5.182 (3.339-8.04) | < 0.001 |  | 4.183 (2.636-6.64) | < 0.001 |

**Abbreviations:** Ref, reference; CI, confidence interval; LVI, lymphovascular invasion; EGC, early gastric cancer; LNM, lymph node metastasis

*** Overlap,** tumor invaded two or more regions simultaneously

Table S2. Clinicopathological characteristics of EGC patients in the training set and the validation set after 1:3 propensity matching

| Parameter | Training set | Validation set | *P-*value |
| --- | --- | --- | --- |
|  | (*n =* 516) | (*n =* 172) |  |
|  | *n* (%) | *n* (%) |  |
| Age |  |  | 0.418 |
| ≤ 60 | 164 (31.8) | 49 (28.5) |  |
| > 60 | 352 (68.2) | 123 (71.5) |  |
| Gender |  |  | 0.088 |
| Male | 320 (62.0) | 94 (54.7) |  |
| Female | 196 (38.0) | 78 (45.3) |  |
| Tumor location |  |  | 0.005 |
| Upper | 120 (23.3) | 28 (16.3) |  |
| Middle | 155 (30.0) | 58 (33.7) |  |
| Lower | 218 (42.2) | 86 (50.0) |  |
| Overlap | 23 (4.5) | 0 (0) |  |
| Tumor size |  |  | 0.719 |
| ≤ 20 mm | 209 (40.5) | 67 (39.0) |  |
| > 20 mm | 307 (59.5) | 105 (61.0) |  |
| Depth of invasion |  |  | 0.134 |
| Mucosa | 266 (51.6) | 100 (58.1) |  |
| Submucosa | 250 (48.4) | 72 (41.9) |  |
| Tumor histological types |  |  | 0.095 |
| Differentiated | 330 (64.0) | 122 (70.9) |  |
| Undifferentiated | 186 (36.0) | 50 (29.1) |  |
| No. of ELNs (mean ± SD) | 29.01± 11.9 | 25.70 ± 11.5 | 0.001 |
| N stage |  |  | 0.120 |
| N0 | 434 (84.1) | 153 (89.0) |  |
| N+ | 82 (15.9) | 19 (11.0) |  |
| Extent of lymphadenectomy |  |  | 0.006 |
| D1 | 55 (10.7) | 28 (16.3) |  |
| D1+ | 80 (15.5) | 39 (22.7) |  |
| D2 | 381 (73.8) | 105 (61.0) |  |
| LNM/LVI |  |  | 0.225 |
| Absent | 420 (81.4) | 147 (85.5) |  |
| Present | 96 (18.6) | 25 (14.5) |  |

**Abbreviations:** SD, standard deviation; LNM, lymph node metastasis; LVI, lymphovascular invasion

Table S3. Comparison of the incidences of LNM/LVI between different risk groups in the training set and validation set after a 1:3 matching

|  | Risk groups | | | | *P*_trend_^a^ |
| --- | --- | --- | --- | --- | --- |
|  | Low-risk | Intermediate-risk | High-risk | Extremely high-risk |  |
| Training set | 5.7% | 15.2% | 22.1% | 41.4% | < 0.001 |
| Validation set | 2.6% | 18.2% | 18.5% | 47.4% | < 0.001 |
| *P*-value^b^ | 0.281 | 0.809 | 0.525 | 0.642 |  |

^a^P value for testing the trend of the incidences of LNM/LVI between different risk groups by study cohorts

^b^P value for testing the difference of the incidences of LNM/LVI between the training set and validation set by risk groups

Table S4. Comparison of the incidences of LNM/LVI between age groups in male and female

|  | Age groups | | *P*-value^a^ |
| --- | --- | --- | --- |
|  | <60 years old | ≥60 years old |  |
| Female | 27.6% | 24.6% | 0.484 |
| Male | 18.5% | 20.6% | 0.393 |
| *P*-value^b^ | 0.004 | 0.268 |  |

^a^P value for testing the difference of the incidences of LNM/LVI between age groups by gender

^b^P value for testing the difference of the incidences of LNM/LVI between male and female by age groups
